# Detection of COVID-19 Based on Chest X-rays Using Deep Learning

**DOI:** 10.3390/healthcare10020343

**Published:** 2022-02-10

**Authors:** Walaa Gouda, Maram Almurafeh, Mamoona Humayun, Noor Zaman Jhanjhi

**Affiliations:** 1 Department of Computer Engineering and Network, College of Computer and Information Sciences, Jouf University, Sakaka 72341, Aljouf, Saudi Arabia; 2 Department of Information Systems, College of Computer and Information Sciences, Jouf University, Sakaka 72341, Aljouf, Saudi Arabia; mfalmufareh@ju.edu.sa (M.A.); mahumayun@ju.edu.sa (M.H.); 3 School of Computer Science and Engineering (SCE), Taylor’s University, Subang Jaya 47500, Selangor, Malaysia; noorzaman.jhanjhi@taylors.edu.my

**Keywords:** COVID-19, chest X-ray, pneumonia, deep transfer learning, neural network (NN)

## Abstract

The coronavirus disease (COVID-19) is rapidly spreading around the world. Early diagnosis and isolation of COVID-19 patients has proven crucial in slowing the disease’s spread. One of the best options for detecting COVID-19 reliably and easily is to use deep learning (DL) strategies. Two different DL approaches based on a pertained neural network model (ResNet-50) for COVID-19 detection using chest X-ray (CXR) images are proposed in this study. Augmenting, enhancing, normalizing, and resizing CXR images to a fixed size are all part of the preprocessing stage. This research proposes a DL method for classifying CXR images based on an ensemble employing multiple runs of a modified version of the Resnet-50. The proposed system is evaluated against two publicly available benchmark datasets that are frequently used by several researchers: COVID-19 Image Data Collection (IDC) and CXR Images (Pneumonia). The proposed system validates its dominance over existing methods such as VGG or Densnet, with values exceeding 99.63% in many metrics, such as accuracy, precision, recall, F1-score, and Area under the curve (AUC), based on the performance results obtained.

## 1. Introduction

Around the world, COVID-19 is wreaking havoc on people’s lives and healthcare systems. It is a new virus strain discovered in 2019 that has never been seen by humans before. The first COVID-19-positive case was discovered in Wuhan, China, in December 2019, and it quickly spread to a number of other Chinese cities as well as several other countries around the world [1,2]. According to preliminary polls, COVID-19 causes minor symptoms in about 99%, while the remainder of cases are serious or critical. The number of people dying from pneumonia caused by the COVID-19 virus is rising every day [3].

**Figure 1:**
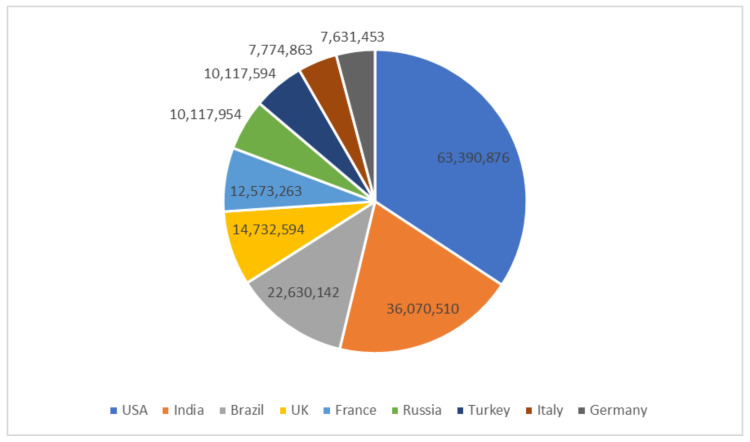
Confirmed COVID-19 cases globally (15 January 2022) [8].

The rapid global spread of COVID-19 put healthcare systems under tremendous pressure; this spread could be significantly slowed if a reliable screening method for patients with COVID-19 infections is established. Doctors and researchers found themselves facing a daunting challenge to find ways to diagnose the disease quickly [4]. A COVID-19 infection can cause serious problems such as acute kidney failure, septic shock, heart attack, and pulmonary edema [5]. The early detection and isolation of patients with infection is critical in combating and addressing the COVID-19 pestilence [6,7]. The prevalence of reported COVID-19 occurrence in the most affected nations around the world is depicted in Figure 1. The United States leads the world in terms of reported illnesses, accounting for 63,390,876 cases out of a total of 185,039,249 cases.

The most common COVID-19 detection technique is real-time polymerase chain reaction (RT-PCR). It has a high percentage of false-negative findings and may take up to two days to receive results, while having a sensitivity range of 70 to 90 [9]; it may also produce a quite high number of false-negative effects and may take up to two days to obtain results. In some countries, it may take up to five days or more due to the overwhelming number of tests that need to be analyzed [4].

Additionally, COVID-19 is detected and diagnosed using radiological screening tests such as CXR and computed tomography (CT). It has been noticed that CXR is one of the most effective methods for diagnosing pneumonia around the world because it is a rapid, inexpensive, and popular clinical method that exposes the patient to less radiation than CTs [10,11].

However, radiologists are needed to look for the radiological signs that show COVID-19 symptoms on a CXR. To save time and effort, it is important to automate the CXR analysis, which is a long and error-prone process that takes a lot of time and effort [12].

**Figure 2:**
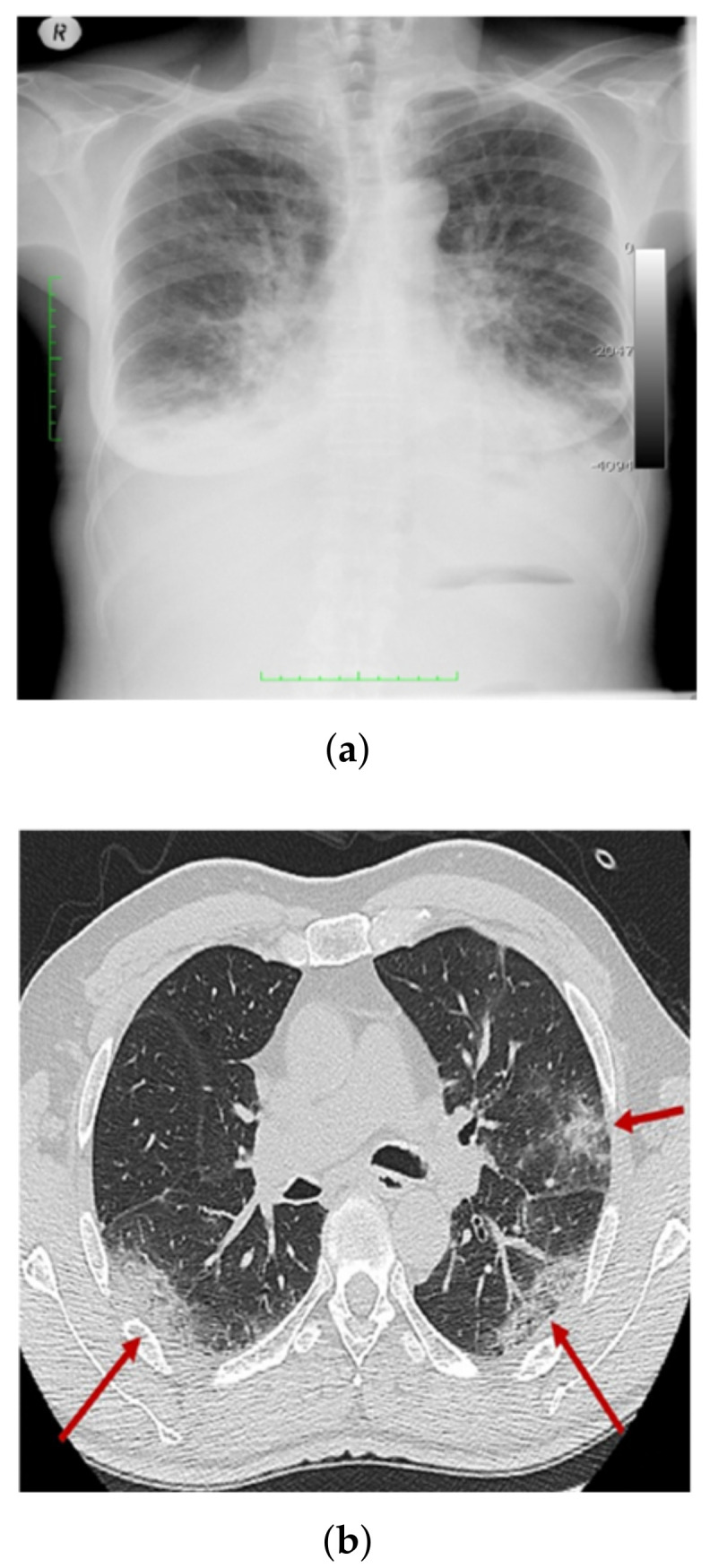
Samples of CXR images and CT images (**a**), and CXR image scan (**b**) CT image scan.

Figure 2 shows a CXR scan image and a CT scan image.

As a result, fully automated and real-time radiography image analyses are required to assist physicians in accurately detecting COVID-19 infection. Physicians may use computer-aided diagnosis (CAD) systems based on DL methods to help them perceive and understand the information in CXR images as well as to overcome the limitations of the imaging acquisition techniques used, rapidly and correctly. DL methods are becoming more common in medical imaging because of their ability to deal with massive datasets that surpass human capabilities. Combining CAD techniques with radiologist medical diagnostics decreases physicians’ stress as well as improves their accuracy and statistical analysis [11].

This paper propose a crossbred DL system for COVID-19 classification and prognosis that uses two unique DL approaches to accurately detect early COVID-19 symptoms from CXR images. The proposed system has two significant phases: preprocessing and classification. The preprocessing phase is used to improve the overall contrast of the image in order to reduce inconsistencies between images obtained from various X-ray devices. The image is also resized and normalized to suit the size of the training model throughout that process. The classification stage, on the other hand, involves a variety of classifiers, and the most effective classifier are chosen based on the classification error for each case.

The following are the key contributions of this research:

1. To determine the feasibility of the proposed scheme, detailed comparative analyses are conducted using various measurement criteria such as accuracy, precision, recall, specificity, F1-score, and AUC.
2. COVID-19 shows radiological indications that are readily detectable on CXR. As a result, DL-based methods can be used to automatically analyze CXR, significantly reducing the analysis time.
3. The COV-PEN dataset is developed, which is a large-scale CXR image dataset. Among those currently publicly available, it includes a large number of CXR images with reported COVID-19 disease.
4. To fine-tune the weights of pre-trained networks on small datasets as well as to train the weights of networks on large datasets, a modified version of Resnet-50 is used.
5. To improve the generalized effectiveness of the suggested method and to prevent over-fitting, a different training protocol assisted by different combinations of training policies (e.g., validation patience and data augmentation) is used.

The following are the remaining parts of this paper: Section 2 summarizes recent related articles, Section 3 explains the methodology used to create the COVID-19 dataset and the proposed system’s design requirements, Section 4 introduces the study findings, and Section 5 ends with the conclusion and possible research opportunities.

## 2. Related Works

This section provided an overview of some related studies for a better understanding of the area under study and to provide the state-of-the-art picture. Convolutional neural networks (CNNs), which are one of the most effective DL models, have successfully proved their mastery over conventional methods in several disciplines, including image classification and pattern recognition [13,14]. Currently, it has indeed been successfully implemented in the field of medicine with impressive outcomes and outstanding performance in different challenging settings. Various medical imaging systems using DL techniques have also been developed to assist physicians and specialists in effective COVID-19 diagnosis, care, and follow-up examination [15,16]. Narin et al. [11] used five-fold cross validation to enforce various binary classifications. With an accuracy equal to 98%, specificity value of 100%, and a recall with 96%, the pre-trained ResNet-50 method gives the best efficiency. On the other hand, Wang et al. [17] have suggested using CXR images to automatically establish a new deep architecture called COVID-Net to detect COVID-19 instances. Using a database containing 13,975 CXR images, this model has the highest classification accuracy of 93.3%. The key strength of this approach is that the conceptual composition could create a balance between different goals such as accuracy and computational costs through architectural design choices. Hemdan et al. [18] introduced COVIDXNet, a DL framework for detecting COVID-19 infections in CXR images. A small dataset of 50 images was used to compare seven DL techniques (e.g., MobileNetV2, ResNetV2, VGG19, DenseNet201, InceptionV3, Inception, and Xception). DenseNet201 had the best performance, with a 91% accuracy score. While Zhang et al. [19] derived useful feature representations from CXR image using ResNet-18 model as a feature vector. Those derived features were then entered as an input into a multi-layer perception. A dataset of hundred images taken from seventy patients yielded the highest accuracy rate of 96%. A further supervised transfer-learning method for COVID-19 infection detection in CXR using an extreme version of the Xception model was developed by Das et al. [12], which achieved accuracy of 97.4%. Furthermore, Ozturk et al. [20] introduced a new system for COVID-19 identification using CXR for automatic detection. It was created to provide consistent and reliable diagnostics for multi-class classifications (COVID-19, mild, and pneumonia) and binary classifications (COVID vs. non-COVID). Using the DarkNet model, they were able to achieve a classification performance of 98.08% for binary classification and 87.02% for the classification of multi-class.

Many studies have tried to find COVID-19 infections in CXR images by using different DL methods [21,22,23,24,25,26,27,28,29,30,31,32,33,34,35,36,37], as indicated in Table 1. The investigation of COVID-19 identification and diagnostic systems that rely on CXR images indicated that there are still a number of vulnerabilities that need additional investigation. For starters, the majority of current systems have been validated with limited CXR datasets as well as a small presence of positive COVID-19 cases. The size of the datasets is insufficient to indicate the true output of the proposed systems. Furthermore, despite the fact that several researchers have achieved high reliability values using pre-trained models through transfer-learning, there has been little focus on developing and training a customized DL model from scratch due to a shortage of a large dataset including a substantial number of CXR images with reported COVID-19 infection.

Eventually, almost all of those studies only concentrated on training DL models used on original images instead of preprocessed images, restricting the capacity of the last classification network to generalize. To address these issues, the current study develops a lightweight COVID-19 detection system that alters the architecture of pre-trained models by inserting several layers, resulting in an optimized proposed system with greater satisfaction.

## 3. Proposed System

**Figure 3:**
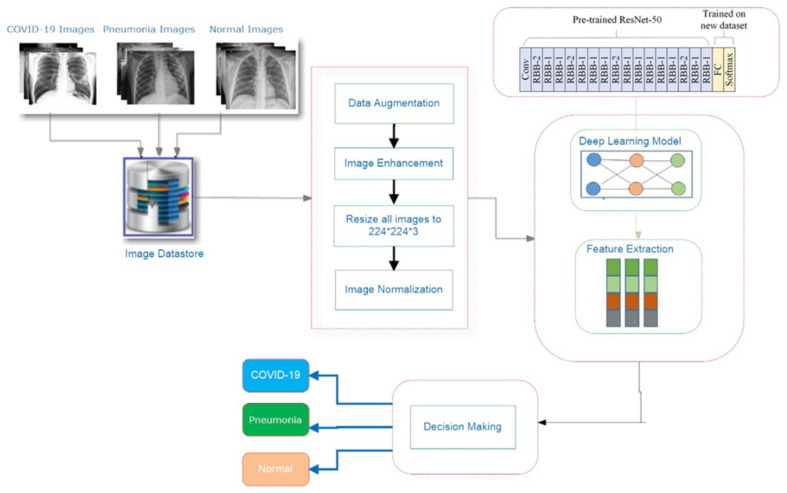
A schematic methodology for the COVID19 detection system.

Figure 3 depicts the schematic methodology for the COVID19 detection system, which requires retraining a transfer DL approach (Resnet-50) over preprocessed images in the image datastore to learn discriminative and useful feature representations as illustrated in Algorithm 1. First, the procedure for constructing the datastore is described briefly. The proposed system’s implementation specifics are then discussed, including the proposed preprocessing algorithms, the main design, and the adopted approach’s training methodology.
**Algorithm 1** Proposed System steps
**Let** ζϵ = chest X-ray, α =Augmentation, pp= pre-Processing, i= image, IEA= Image enhancement algorithm, r= rotation, s=scaling, rf=reflection, sm= shifting methods**Step 1:** Read( ζϵ )**Step 2:** α(image) w.r.t. r, s, rf, sm**Step 3:** Perform (pp (i))    3.1. Apply(IEA)    3.2. Resize (i)/224*24*3    3.3. Normalize pixelvalue(i)/interval [0, 1]        3.3.1. Conversion        3.3.2. Calculation (mean)        3.3.3. Scaling(i)        3.3.4. Convesrionback    3.4. Split (dataset)/training, testing    3.5. Extract(features)/Resnet-50 pre-trained model    3.6. Optimize (Freeze layers, epochs, learning weights,    batch size)/optimization methods**Step 4:** Calculate VPM (accuracy, confusion matrix, ROC, AUC, Precision, recall, F1)**Step 5:** Comparison (recent studies)


### 3.1. COV-PEN Image Datasets

Data are at the heart of DL, and it is used as a fuel for these learning models. COVID-19 is a novel disease, and a plethora of datasets are currently available. In this work, we gathered CXR images from two publicly accessible image databases of reported infected cases to create a dataset. There are 2790 CXR images in the dataset used to train and test the proposed system, which we refer to as COV-PEN. To build the COV-PEN dataset, we combined two publicly available data repositories:

1. COVID-19 Image Data Processing [41].
2. CXR Images (Pneumonia) [42].

**Figure 4:**
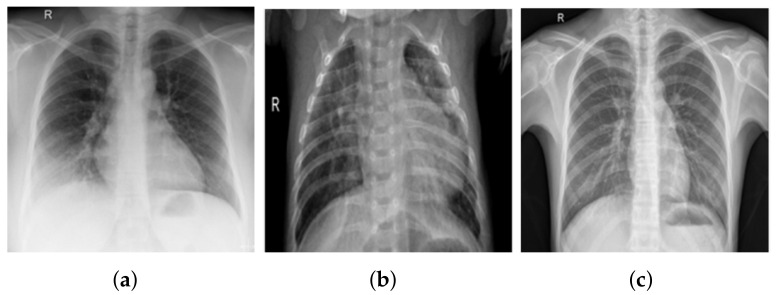
CXR images from COV-PEN dataset: (**a**) COVID-19, (**b**) pneumonia, and (**c**) mild.

COVID-19 X-ray files were created by Joseph et al. [41] and are available in an open source Github repository. The authors gathered radiology photographs from various authentic records of COVID-19 incidents for analysis purposes, and most COVID-19 studies use images from this source. A free archive of COVID-19 cases of CXR or CT images is available in the registry, which is maintained on a regular basis. At the time of publication, the archive had about 930 COVID-19 chest radiography files. Pneumonia and standard CXR images were gathered from the Kaggle list “CXR Images (Pnemonia)” [42]. There are 1583 mild cases and 4273 pneumonia cases in the dataset. The COV-PEN dataset, in particular, includes 2790 CXR images from these two sources. Figure 4 summarizes some CXR image samples from the COV-PEN dataset, showing the variety of patient cases in the dataset. Since both databases are open access and publicly accessible to the scientific community and the general public, they were chosen to create COV-PEN.

### 3.2. Image Preprocessing Step

**Figure 5:**
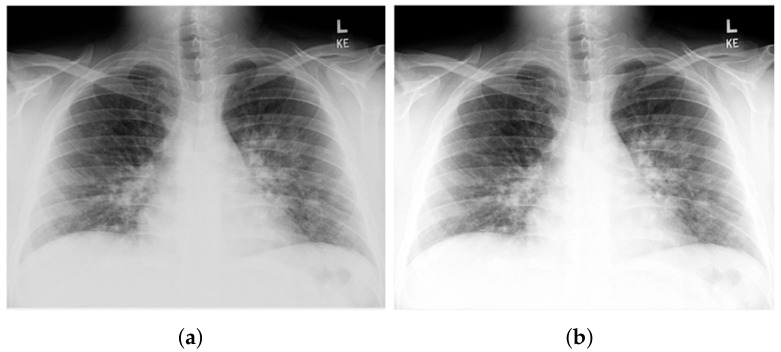
Output of the proposed image enhancement process: (**a**) raw CXR image and (**b**) enhanced image.

This step includes data augmentation, image enhancement, image rescaling, and normalization, among other things. Since the model’s network becomes more sophisticated, the number of parameters to learn increases as well, leading to overfitting. After dividing the COV-PEN dataset into three mutually exclusive sets (e.g., preparing, verification, and evaluating sets) to overcome the overfitting issue created by the small number of training photos, data augmentation was used to prevent skewed prediction outcomes. Augmented images with corresponding masks such as rotation, reflection, shifting, and scaling are generated for each image in the dataset. The accuracy of a raw CXR image produced by an electronic detector is simply inadequate, reducing the availability for detection and diagnosis. To improve the quality of CXR images, image enhancement techniques should be used. Furthermore, training deep neural networks (DNNs) on top of preprocessed images rather than raw image data will significantly reduce the DNNs’ generalization error and training time. As a result, an appropriate image enhancement technique was proposed to improve the low quality of the CXR image before feeding it into the proposed system. First, the CXR image’s small details, textures, and low contrast were improved using adaptive contrast enhancement based on redistribution of the input image’s lightness values by taking the image as an input and to give out an enhanced image based on redistributing the histogram of the image, as shown in Figure 5. As a consequence, this approach improves the visibility of the edges and curves in each part of an image while also enhancing the image’s local contrast. Since the images in the dataset come from multiple datasets and could also come from various cameras, the parameters of image acquisition often vary because a portion of images have small pixel sizes and all of the images must be rescaled. Therefore, there are significant changes in the image’s brightness and size. Moreover, the images in the Kaggel dataset are grayscale; we must replicate the image three times to obtain an RGB image. Most of the images in the CXR image dataset almost certainly originated from various acquisition devices, each with its own set of requirements. The intensity of the pixel of each image can vary significantly, so the pixels intensity of all images is normalized between [−1, 1] to ensure that the data are within specific ranges and noise is removed. Normalization has the benefit of ensuring that the model is less vulnerable to slight variations in weights, making it easier to optimize.

### 3.3. Proposed Transfer Learning for COV-PEN Detection

**Figure 6:**
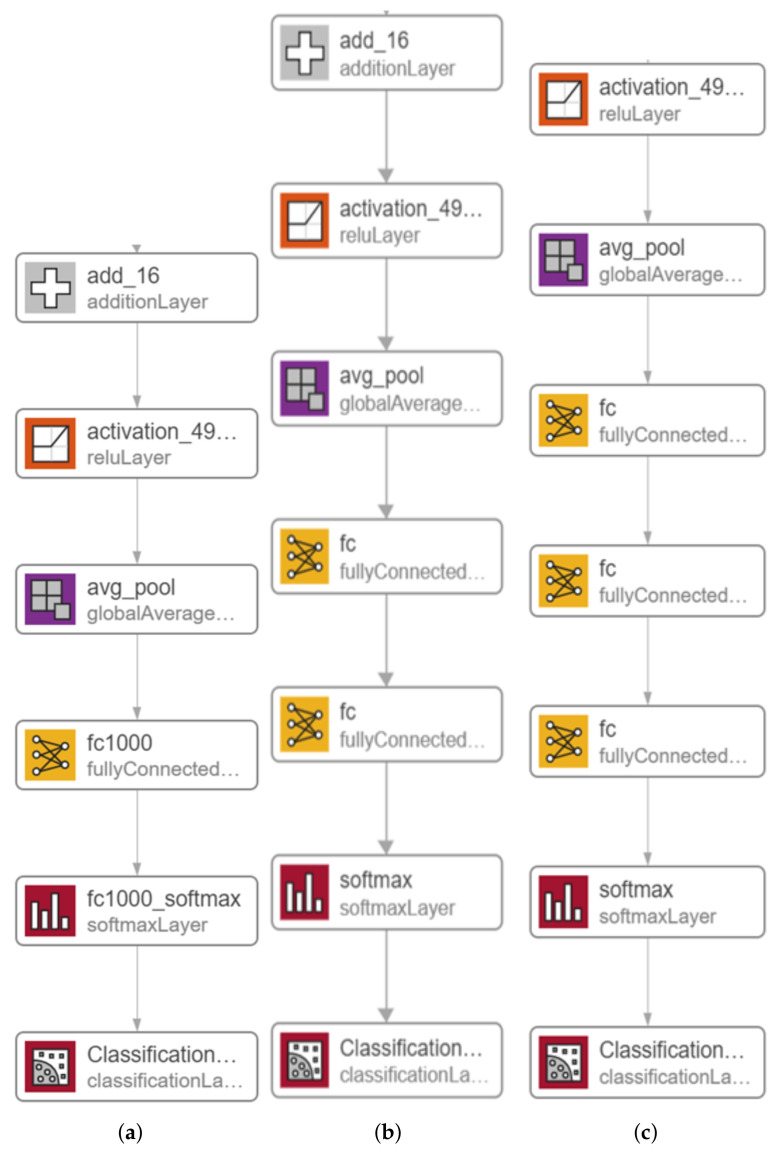
Modified versions of the proposed Resnet-50 model: (**a**) original pre-trained model, (**b**) adding one FC layer, and (**c**) adding two FC layers and one Sigmoid.

The proposed system’s main architecture is based on the Resnet-50 model. The massive number of structures and hyper-parameters to be determined is the most difficult challenge when using DL models (e.g., learning rate, number of batch size, number of frozen layers, and number of epochs, etc.). The effects of various hyper-parameters value on the performance of the proposed systems is investigated. In this section, we describe in detail the potential solution based on a modified version of one of the Resnet-50 [43] model. In 2015, He K. et al. [43] developed Resnet-50, a residual learning component to the CNN architecture. A standard layer with a skipped connection compensates the residual unit. The skip connection enables a layer’s input signal to traverse the network by linking it to that layer’s output. As a result of the residual units, an extremely deep 152-layer model was trained, which won the 2015 LSVRC2015 competition. Its innovative residual structure allows for a more straightforward gradient flow and more efficient training. It has a top-five error rate of less than 3.6 percent. ResNet has 34, 50, and 101 layers in other versions. In this work, we investigate two modified versions of the Resnet-50 model as well as the original model, which are illustrated in Figure 6. The original Resnet-50 model is shown in Figure 6a. In order to build the proposed two versions, we modify the latest layers by adding one fully connected (FC) Layer with a size of 512 and two FC layers with sizes of 2048 and 1024. We also replace the original FC layer and softmax layer in both versions, as shown in Figure 6b and Figure 6c, respectively. The original layers of the Resnet-50 model are pre-trained on the ImageNet dataset [44]. Consequently, initially, the new added layers are assigned random weights. Then, during training, all model weights are updated using the back-propagation algorithm, which is the main algorithm for training neural network models.

Experiments using Resnet-50 without adding an additional FC was performed and the results were very poor, so that we took this trend of adding more FC at the end of the Resnet. In the first modified version of Resnet-50, shown in Figure 6b, the first FC was replaced with a new FC layer with a size of 512 and one FC layer with a size of 3; the number of classes was added after the replaced FC layer and before softmax layer, which also has been replaced with a new softmax layer. Based on what was mentioned by Basha, S.S et al. [45], when dealing with small datasets, the network needs more FC layers than when dealing with larger datasets. Any neuron from the previous layer is connected to every other neuron in the next layer in the fully connected layer, and each value contributes to predicting how well a value fits a given class. The output of the final FC layer is then redirected to an activation function, which calculates the class scores. One of DNN’s most common classifiers is Softmax which computes the probability distribution of the n output groups through its equations. The only drawback to adding a single FC layer is that it is extremely computationally intensive.

In the second modified version depicted in Figure 6c, we added two FC layers connected between the first FC layer and the softmax layer, which also has been replaced by a new FC layer and a new softmax layer, respectively. The size of the first FC layer is 2048, the size of the second FC layer is 1024, and the size of the third FC layer is 3. We use batch normalization because it is effective at combating network overfitting because of the fact that overfitting occurs when the model learns the training data extremely well but does not generalize well to other testing data. It is a common problem in DL models, and the risk of falling into this problem increases in situations where the training dataset is small, which is the case in this study. DNNs algorithms always produce results with a degree of variability [46] because, in such algorithms, many steps involve a degree of randomness. Thus, one way to improve the performance of DNNs algorithms is to use ensemble learning. Ensemble has multiple definitions, one of this definitions used here is to run the network for n times using the same network parameters (epochs, batch size, optimizer, etc.). In our work, we propose to implement stacked generalization by performing multiple training runs of the same model, which we refer to this as the multiple-runs ensemble.

## 4. Experimental Results

Several sufficiently large experiments were performed on the COV-PEN dataset to demonstrate the efficiency of the proposed DL systems and to equate their results to the existing state-of-the-art approaches. The proposed system’s code was written throughout MATLAB R2020b and evaluated on a Windows 10 machine with a Core i7-4650U CPU and 8 GB of RAM. All tests were carried out using an 80 percent random array of CXR images as a training collection for the proposed DL systems, according to the proposed training scheme. During the learning process, ten percent of the training data were chosen at random and used as a validation set to assess their abilities and to save the weight combinations with the highest accuracy value. The proposed framework is pre-trained on the COV-PEN dataset using the Adam and sigmoid optimizer with a learning rate strategy that decreases the learning rate when learning becomes stagnant for a period (i.e., validation patience). The following hyper-parameters were used for training in the Adam optimizer: Number of epochs = 15; batch size varying from 32 to 128, with a move of double its previous value; patience = 6; loss function = categorical cross-entropy; and momentum= 0.95. Finally, we incorporate a batch re-balancing strategy to enhance infection form distribution at the batch stage.

### 4.1. Assessment Methods

To evaluate performance, we compared our proposed system with other systems using the performance metrics listed in Equations (1)–(4),:

(1) $$\;\mathrm{Accuracy}\;=\;\frac{\mathrm{TP}+\mathrm{TN}}{\mathrm{TP}\;+\;\mathrm{TN}\;+\;\mathrm{FP}\;+\;\mathrm{FN}}$$

(2) $$\;\mathrm{Precision}\;=\;\frac{\mathrm{TP}}{\mathrm{TP}\;+\;\mathrm{FP}}$$

(3) $$\mathrm{Sensitivity}\;=\;\mathrm{Recall}\;=\;\frac{\mathrm{TP}}{\mathrm{TP}\;+\;\mathrm{FN}}$$

(4) $$F1-\mathrm{Score}=\frac{2\;\times \;\mathrm{Precision}\;\times \;\mathrm{Recall}}{\mathrm{Precision}+\mathrm{Recall}}=\frac{2\;\times \;\mathrm{TP}}{2\;\times \;\mathrm{TP}\;+\;\mathrm{FP}\;+\;\mathrm{FN}}$$

Here, TP denotes true positives (patients correctly identified as having COVID-19), TN denotes true negatives (patients correctly identified as not having COVID-19), FP denotes false positives (patients with lung diseases other than COVID-19 or mild lung identified as having COVID-19), and FN denotes false negatives (patients with COVID-19 identified as not having the disease).

### 4.2. Results of the Proposed Systems

In this section, we report the different experiments’ results of the proposed systems using the COV-PEN dataset with a 80–20% train–test split. That split is selected to ensure that execution times were not prohibitive. In the first experiment, we trained the Resnet-50 first version and second version models for 10 epochs using 10% of the training set as a validation set, a batch size of 128, and a learning rate ranging from 0.0002 up to 0.001 and froze the weights of the first 50 layers of the model. We executed the training three times and monitored the average accuracy measures over the validation set. Table 2, Table 3, Table 4 and Table 5 show the average accuracy of an ensemble of the modified models. As mentioned previously, we built the model ensemble using multiple runs (three runs for the same parameters) to train the same model with the same parameters (Runs 1–3, Table 2, Table 3, Table 4 and Table 5). An observation can be made regarding the accuracy, which varies from run to run as the weights are initialized randomly each run; only the best run result is saved. Comparing the two versions, the best achieved accuracy for the first and second version models are 97.84% and 94.26%, respectively.

**Figure 7:**
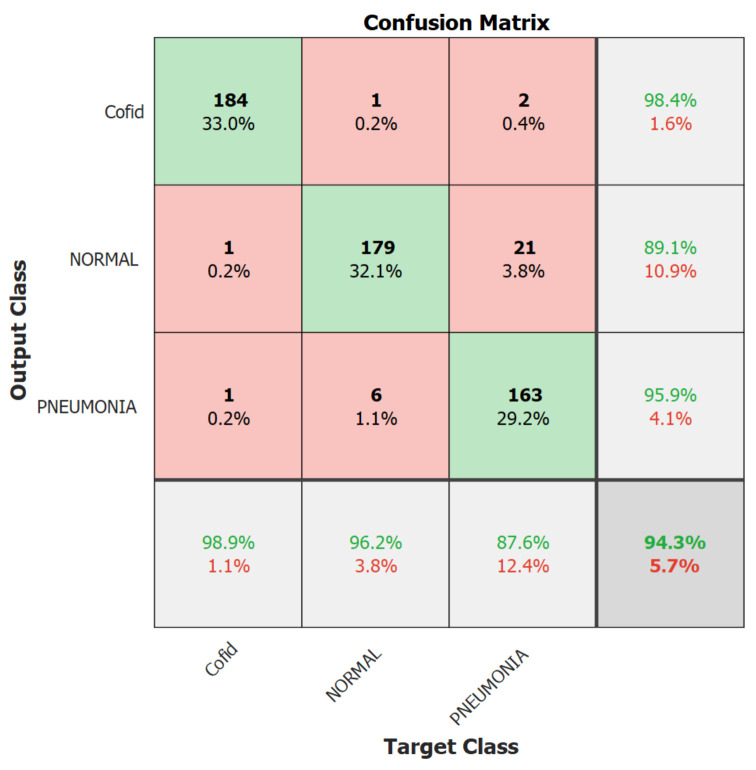
The best result’s confusion matrix for the first version model freeze = 0.

**Figure 8:**
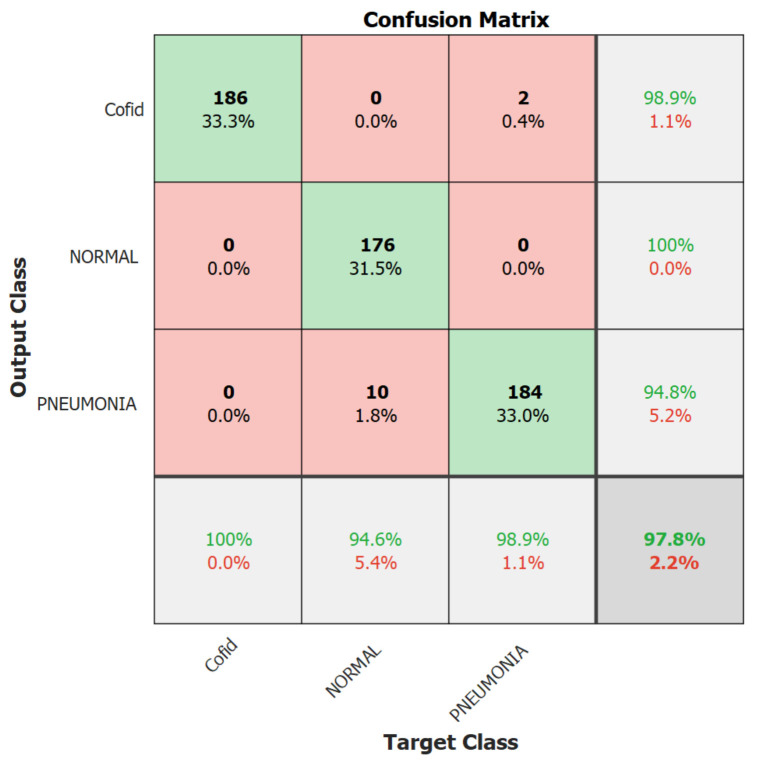
The best result’s confusion matrix for the first version model freeze = 50.

**Figure 9:**
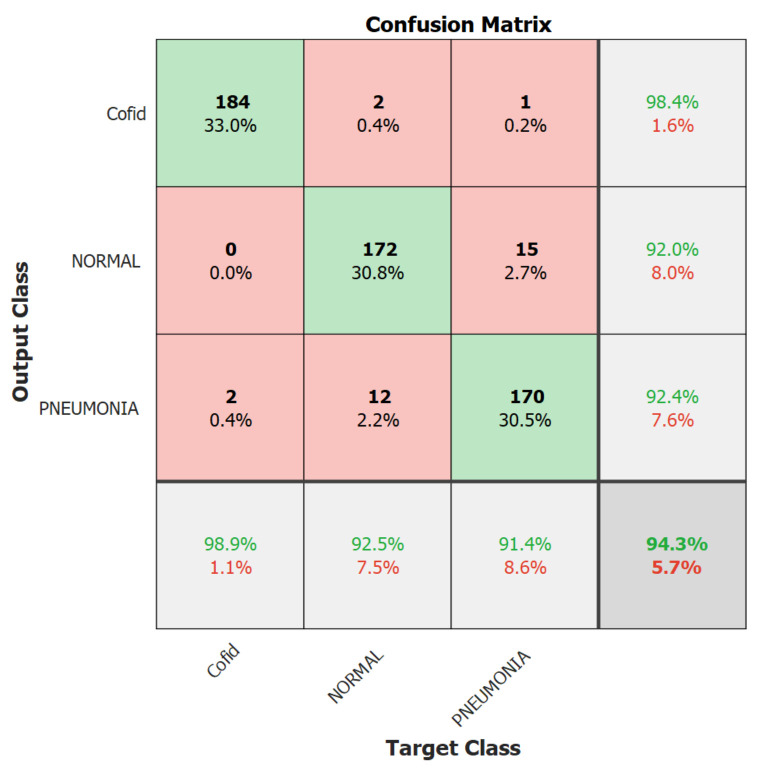
The best result’s confusion matrix for the second version model.

We focus on the first version model in the second experiment because it outperformed the second version model. Therefore, in the second experiment, we trained the Resnet-50 first version model only for 10 epochs using 10% of the training set as a validation set, a batch size of 128, and a learning rate ranging from 0.0002 up to 0.001 with no freezing for the weights. We executed the training three times and monitored the average accuracy measures over the validation set. Table 6 and Table 7 show the average accuracies of an ensemble of the first version model, which are 86.63% and 94.24% using the Adam and sgmd optimizers, respectively. Eventually, Figure 6, Figure 7 and Figure 8 demonstrate the three confusion matrices of COVID-19 infected and non-COVID-19 test results using the first version model with freeze = 0 and freeze = 50 and the second version model with freeze = 50, respectively. Figure 7 indicates that two COVID-19-infected images were misclassified as non-COVID-19 images while two non-COVID-19 images were misclassified as COVID-19 images, and Figure 8 reveals that two COVID-19-infected images were misclassified as non-COVID-19 images whereas no non-COVID-19 images were misclassified as COVID-19 images. Finally, Figure 9 shows that three COVID-19-infected images were misclassified as non-COVID-19 images, and two non-COVID-19 images were misclassified as COVID-19 images.

**Figure 10:**
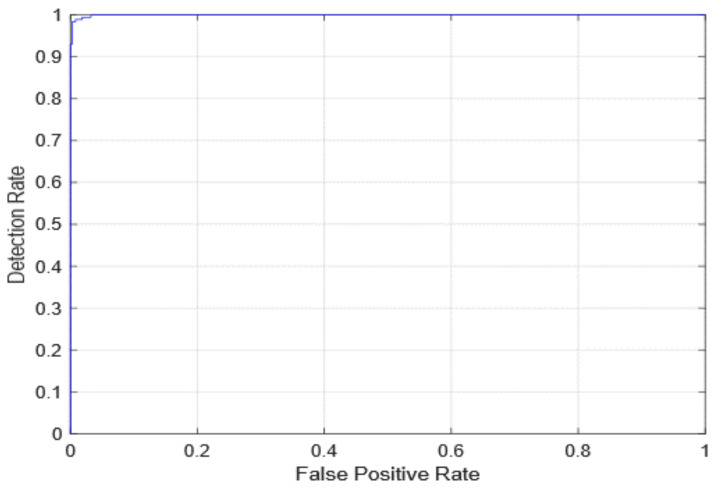
ROC curve for the first version model freeze = 0.

**Figure 11:**
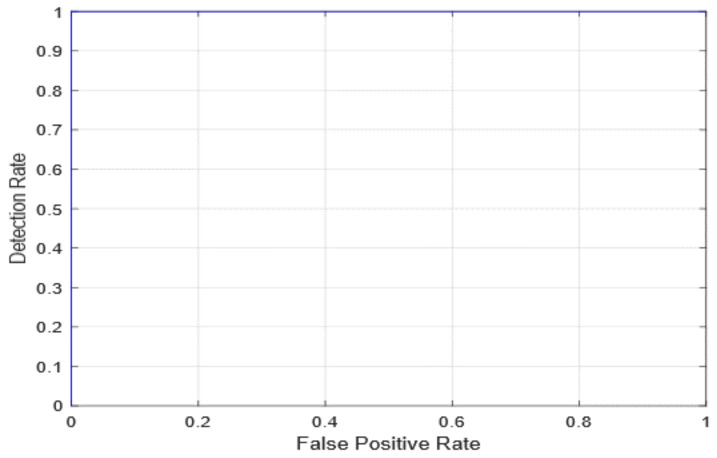
ROC curve for the first version model freeze = 50.

**Figure 12:**
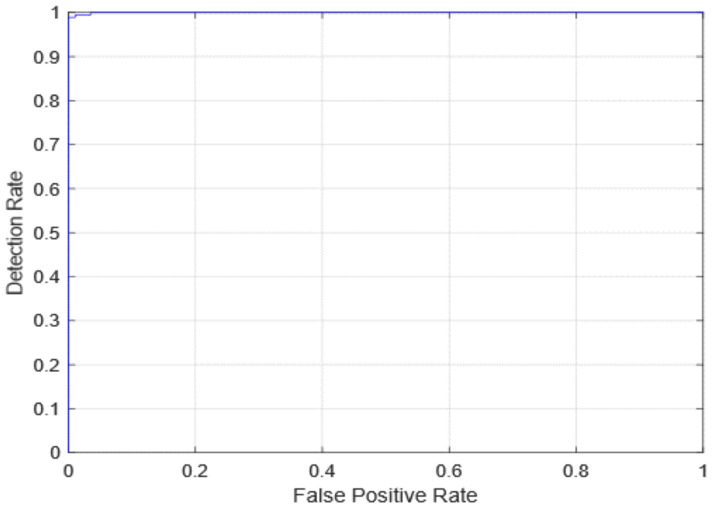
ROC curve for the second version model.

The AUC receiver operating characteristic (ROC) curve, which is a graph that shows a model’s classification performance on two parameters: true positives and false positives, was used to evaluate diagnostic efficiency. By incorporating the areas of small trapezoidal fragments under the ROC curve, the AUC can be determined. The proposed method’s AUC evaluations as well as the first edition (with freeze = 0 and freeze = 50) and 2nd version models are seen in Figure 10 and Figure 11. In Figure 12, the AUC is also similar, which is the best-case scenario. The proposed first edition framework classification model (with AUC = 1) performs slightly better than existing COVID-19 classification models.

Table 8 shows the best results obtained using the first version with freeze = 0 and freeze = 50 and for the second version models. The disparity in performance can be explained by the fact that the first version with freeze = 0 and the second version models have an extra layer that starts with random weights rather than pre-trained weights, as in the other layers. These random weights add extra degrees of freedom to the model and should improve its generalization ability.

**Figure 13:**
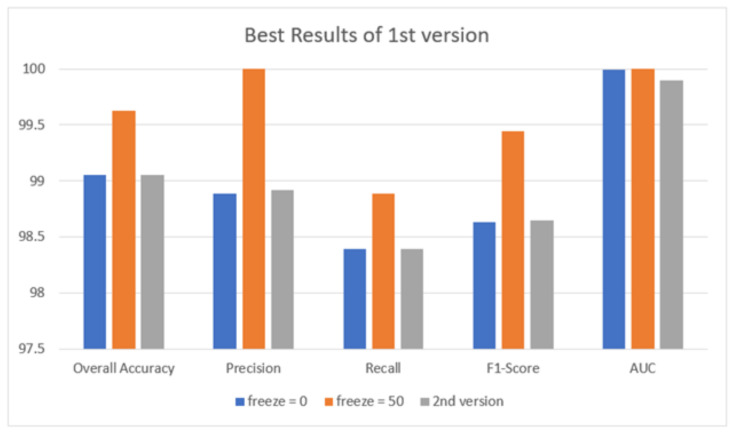
Best result for all models.

Figure 13 illustrates the best results obtained in terms of overall accuracy, precision, recall, F1-score, and AUC. It reveals that the best result is obtained when using the first version model and freeze = 50.

The first version model is used in the efficiency comparison of the proposed COV-PEN scheme with current state-of-the-art approaches, in addition to its usefulness in leveraging the great strengths of each classifier. These findings bolstered the case for implementing the proposed COV-PEN method in real-world environments to help radiologists diagnose COVID-19 infection more accurately using CXR images while also reducing their workload.

### 4.3. Comparison with State-of-the-Art Methods

**Figure 14:**
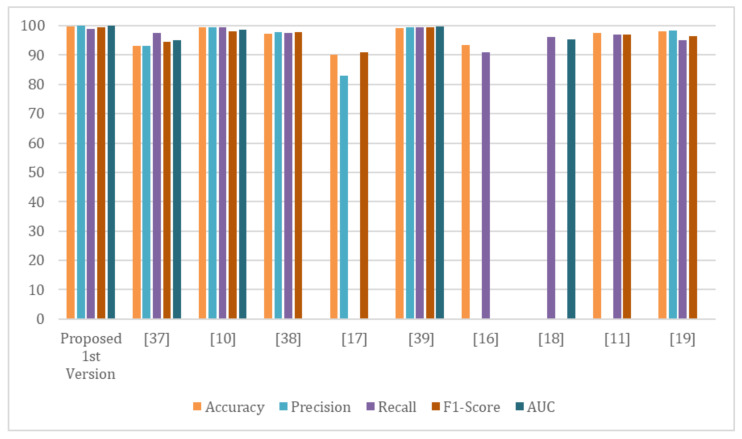
The proposed first version model performance in comparison with current systems.

The proposed system’s performance and reliability are compared with the most recent state-of-the-art COVID-19 detection systems. In this section, we present the proposed first version system’s outcomes and compare them with existing methods (see Table 9). As revealed in Figure 14, the proposed system demonstrates remarkable results that are more accurate than existing methods. Furthermore, compared with other models such as VGG16 or DenseNet, the proposed improved Resnet-50 system is lightweight. In terms of precision, accuracy, sensitivity, and F1-score, our proposed system outperformed existing methods.

## 5. Conclusions and Future Work

A reliable and automatic mechanism for COVID-19 diagnosis is presented using chest radiography images to differentiate between patients with mild, pneumonia, and COVID-19 infections. Image enhancement techniques were used in the proposed system to improve the intensity of the CXR image and to eliminate noise. To avoid overfitting and to improve the overall capabilities of the proposed DL systems, two different DL approaches (first and second proposed versions of Resnet-50) were trained on top of preprocessed chest medical imaging. To evaluate the reliability of the proposed system, a CXR image dataset labeled the COV-PEN dataset was built. With an overall accuracy of 99.63%, a precision of 100%, a recall of 98.89%, an F1-score of 99.44%, and an AUC of 100%, the proposed system performs equally well for expert radiologists. The proposed system outperforms current models, per the comparative studies. Experiments with a huge and challenging dataset containing multiple COVID-19 cases are required to demonstrate the efficacy of the proposed system. Other methods, such as Densenet, VGG, or Inception-Resnet network, may be used on the COV-PEN dataset as a future work. 

## Figures and Tables

**Table 1:** Literature comparison of COVID-19 diagnostic methods using CXR images.

Recent Work	Techniques Used	Number of Classes	Accuracy
Khan et al. [21]	CoroNet	4	89.6%
Ucar and Korkmaz [22]	Bayes-SqueezeNet	3	98.3%
Apostopolus et al. [23]	VGG-19	3	93.48%
Sahinbas & Catak [24]	VGG-16, VGG-19, ResNet, DenseNet, InceptionV3	2	80%
Jamil and Hussain [25]	Deep CNN	2	93%
Alzab et al. [26]	VGG-16	2	-
Joaquin. [27]	ResNet-50	2	96.2%
Sethy et al. [28]	ResNet-50 + SVM	3	95.33%
Houssein et al. [29]	hybrid quantum classical CNNs	3	88.6%
Saad et al. [30]	CNN, GoogleNet, ResNet-18	2	99.3%
Apostolopoulos, & Mpesiana [31]	MobileNetV2	3	96.78%
Oh et al. [32]	ResNet-18	3	88.9%
Brunese et al. [33]	VGG-16	3	96%
slam et al. [34]	CNN+LSTM	2	99.4%
Ezzat et al. [35]	DenseNet121+GSA	2	98.3%
Sahlol et al. [36]	Inception + FO-MPA	2	99.6%
Toraman et al. [37]	Capsule Network	2	97.24%
Rajaraman, S. and Antani, S. [38]	VGG16	2	93.0%
Afshar, P. et al. [39]	capsule network	2	97.2%
Elshennawy, N. & Ibrahim, D. [40]	ResNet152V2, MobileNetV2	2	99.22%

**Table 2:** Average accuracy for the first version model using the COV-PEN dataset with the first 50 layers frozen, epochs = 15, optimizer = Adam, and batch size = 128.

Learning Rate	Ensemble Using Several Runs		
	Run 1	Run 2	Run 3
0.0002	0.949820789	0.924731183	0.955197133
0.0004	0.935483871	0.919354839	0.928315412
0.0006	0.964157706	**0.978494624**	0.935483871
0.0008	0.956989247	0.930107527	0.944444444
0.001	0.935483871	0.919354839	0.928315412

**Table 3:** Average accuracy for the first version model using the COV-PEN dataset with the first 50 layers frozen, epochs = 15, optimizer = sgmd, and batch size = 128.

Learning Rate	Ensemble Using Several Runs		
	Run 1	Run 2	Run 3
0.0002	0.953405018	0.955197133	**0.962365591**
0.0004	0.931899642	0.910394265	0.949820789
0.0006	0.949820789	0.948028674	0.931899642
0.0008	0.919354839	0.944444444	0.9390681
0.001	0.931899642	0.910394265	0.949820789

**Table 4:** Average accuracy for the second version model using the COV-PEN dataset with the first 50 layers frozen, epochs = 15, optimizer = Adam, and batch size = 128.

Learning Rate	Ensemble Using Several Runs		
	Run 1	Run 2	Run 3
0.0002	0.853046595	0.903942652	0.808960573
0.0004	0.749820789	**0.907526882**	0.892473118
0.0006	0.678136201	0.808960573	0.747311828
0.0008	0.88781362	0.62437276	0.716845878
0.001	0.683512545	0.679928315	0.617845867

**Table 5:** Average accuracy for the second version model using the COV-PEN dataset with the first 50 layers frozen, epochs = 15, optimizer = sgmd, and batch size = 128.

Learning Rate	Ensemble Using Several Runs		
	Run 1	Run 2	Run 3
0.0002	0.933691756	0.937275986	0.933691756
0.0004	0.939068100	0.935483871	0.903942652
0.0006	**0.942652329**	0.917562724	0.907526882
0.0008	0.892473118	0.716845878	0.808960573
0.001	0.747311828	0.921146953	0.808960573

**Table 6:** Average accuracy for the first version model using the COV-PEN dataset freeze = 0, epochs = 15, optimizer = Adam, and batch size = 128.

Learning Rate	Ensemble Using Several Runs		
	Run 1	Run 2	Run 3
0.0002	0.815412186	0.843010753	0.842175627
0.0004	0.607526882	0.772401434	0.607526882
0.0006	**0.866308244**	0.798207885	0.81172043
0.0008	0.610394265	0.678853047	0.607526882
0.001	0.756630824	0.733333333	0.734050179

**Table 7:** Average accuracy for the first version model using the COV-PEN dataset freeze = 0, epochs = 15, optimizer = sgmd, and batch size = 128.

Learning Rate	Ensemble Using Several Runs		
	Run 1	Run 2	Run 3
0.0002	**0.94265233**	0.937275986	0.933691756
0.0004	0.933691756	0.908602151	0.919002151
0.0006	0.9390681	0.88172043	0.843010753
0.0008	0.935483871	0.869175627	0.81172043
0.001	0.734050179	0.790322581	0.756630824

**Table 8:** Best result for all models.

Quantitative Measures	1st Version Model (freeze = 0)	1st Version Model (freeze = 50)	2nd Version Model
Overall Accuracy	99.05	**99.63**	99.05
Precision	98.89	**100**	98.92
Recall	98.39	**98.89**	98.39
F1-score	98.63	**99.44**	98.65
AUC	99.99	**100**	99.98

**Table 9:** Comparison of the proposed 1st version model with existing systems.

Reference	Dataset	Classifier	Accuracy	Precision	Recall	F1-Score	AUC
Proposed 1st version	COV-PEN	Modified version of Resnet-50	99.63	100	98.89	99.44	100
Afshar, P. et. al. [39]	- COVID-19 Image Data Collection	—	97.2	97.67	97.5	97.70	—
	- Chest X-ray Images (Pneumonia)						
	- COVID-19 Image Data Collection						
Rajaraman, S. and Antani, S. [38]	- Pediatric CXR dataset	VGG16	93.0	93.15	97.53	94.57	95.0
	- RSNA CXR dataset	InceptionV3					
	- CheXpert CXR dataset	Xception					
	- NIH CXR-14 dataset	Densenet121					
	- Twitter COVID-19 CXR dataset	NASNet-Mobile					
	- COVID-19 Image Data Collection						
Narin, A., et. al. [11]	- Chest X-ray Images (Pneumonia)	ResNet-50	99.5	99.4	99.5	98.0	98.7
	- COVID-19 Image Data Collection	ResNet-101					
		ResNet-152					
		InceptionV3					
		InceptionRes					
		net-V2					
	- Chest X-ray Images (Pneumonia)						
Hemdan, E. et. al. [18]	COVID-19 Image Data Collection	DenseNet201	90	83	—	91.00	—
Elshennawy, N. & Ibrahim, D. [40]	Chest X-ray Images (Pneumonia)	ResNet152V2	99.22	99.43	99.44	99.44	99.77
		MobileNetV2					
Wang et al. [17]	- COVID-19 Image Data Collection	VGG-19	93.3	—	91	—	—
	- COVID-19 Chest X-ray Dataset	Resnet-50					
	- ActualMed COVID-19 Chest X-ray Dataset	COVID-Net					
	- RSNA Pneumonia Detection Challenge dataset						
	- COVID-19 radiography database						
Zhang et al. [19]	- COVID-19 Image Data Collection	ResNet-18	—	—	96	—	95.18
	- Chest X-ray Images (Pneumonia)						
Das et al. [12]	- COVID-19 Image Data Collection	extreme version of the Inception (Xception) model	97.40	—	97.09	96.96	—
	- ChestX-ray8 database (Pneumonia	Normal)					
Ozturk et. al. [20]	- COVID-19 Image Data Collection	DarkNet	98.08	98.3	95.1	96.5	—
	- ChestX-ray8 database (Pneumonia	Normal)					

## Data Availability

Publicly available datasets were analyzed in this study. This data can be found here: [https://www.kaggle.com/paultimothymooney/chest-xray-pneumonia (accessed on 13 December 2021); https://github.com/ieee8023/covid-chestxray-dataset/tree/master/images] (accessed on 13 December 2021).
